# Will Rogers revisited: prospective observational study of survival of 3592 patients with colorectal cancer according to number of nodes examined by pathologists

**DOI:** 10.1038/sj.bjc.6603352

**Published:** 2006-09-12

**Authors:** S George, J Primrose, R Talbot, J Smith, M Mullee, D Bailey, C du Boulay, H Jordan

**Affiliations:** 1 Public Health Sciences and Medical Statistics, Southampton General Hospital, University of Southampton, Mailpoint 805, Tremona Road, Southampton SO16 6YD, UK; 2 F Level Centre Block (MP816), Southampton General Hospital, University Surgery, Tremona Road, Southampton SO16 6YD, UK; 3 Department of Surgery, Poole Hospital, Longfleet Road, Poole BH15 2JB, UK; 4 Hampshire and Isle of Wight Strategic Health Authority, Oakley Road, Southampton, SO16 4GX, UK; 5 South and West Cancer Intelligence Service, Highcroft, Romsey Road, Winchester, Hampshire, UK; 6 Department of Pathology, Southampton General Hospital, University of Southampton, Mailpoint 813, Tremona Road, Southampton SO16 6YD, UK

**Keywords:** colorectal cancer, stage migration, lymph nodes

## Abstract

To investigate the relationship between survival in colorectal cancer patients and the number of lymph nodes examined by a pathologist, previously attributed to stage migration, we used data from a cohort of 5174 colorectal cancer patients recruited between September 1991 and August 1994, and followed-up for 5 years. We selected cases with data present on all prognostic variables, and stratified them into three groups by number of nodes examined. We made a multivariate survival comparison using a Cox regression model. In all, there were 3592 cases with data present on all prognostic variables. Patients who had >10 nodes identified had a significant survival advantage over those who had 5–10 identified, who had in turn a similar advantage over those with 0–4 identified (*P*<0.001). This effect was present in the whole group and at all Dukes' stages, although statistically significant only in stages B (*P*=0.004) and C (*P*=0.019). The effect remained after adjustment in a Cox regression model in which the mean number of nodes taken out by each surgical firm did not predict survival. In a sub-group with data on lymphocytic infiltration into the primary tumour a survival advantage was noted in those with prominent rather than mild infiltration (*P*<0.001): the former also tended to have more nodes found (*P*=0.015). Stage migration alone cannot explain these results, as survival advantages are noted across the whole population independent of stage. Lymphocytic infiltration into the primary tumour is prognostically important, and is associated with the number of nodes found. Reactive enlargement of lymph nodes in the mesentery may make them easier to find, reflect immune response to the tumour, and thus indirectly impact upon survival.

Colorectal cancer is a common malignancy in the western world, second in frequency only to lung cancer when figures for males and females are combined (Schottenfeld and Winawer, 1996). There are around 30 000 incident cases in the UK every year, and more than 150 000 in the USA. Surgery remains the primary treatment in the majority of patients but about half those who undergo surgery develop incurable recurrent disease. Survival from colorectal cancer depends upon the tumour stage at presentation and most patients are in Dukes stage B or C (UICC stages II and III) at diagnosis (Williams *et al*, 1995).

Several groups have observed a relationship between the number of lymph nodes identified at pathological examination and survival in colorectal cancer patients, examining usually Dukes' B, or Dukes' B and C patients (Michiels *et al*, 1994; Caplin *et al*, 1998; Tepper *et al*, 2001; Cserni *et al*, 2002; Goldstein, 2002; Prandi *et al*, 2002; Swanson *et al*, 2003; Jestin *et al*, 2005). All groups have concluded that the observed improvement in survival was an artefact brought about by inadequate laboratory technique: that is, had more nodes been examined, some of those staged as Dukes' B would have been found to be Dukes' C patients, with consequently poorer survival. Another study demonstrated that the proportion of nodes identified as positive increased along with the number examined, but did not examine survival (Maurel *et al*, 1998). In this paper, we examine evidence to support the hypothesis that the effect of number of nodes harvested on survival is explained by stage migration, using prospective data from a large UK population based cohort of colorectal cancer patients.

## METHODS

This study was carried out in the former Wessex health region of South West England. When data collection began in 1991, Wessex had a population of just over 3 million. It has a substantial rural population and a socio-economic profile that is slightly more affluent than the UK average.

All patients with a new diagnosis of colorectal cancer were prospectively recorded between September 1991 and August 1994. Staff based in hospitals around the region followed standard protocols to extract data from medical records and the data collection identified 5176 cases of colorectal cancer. Data were checked against routine cancer registry records to ensure completeness, and included patients referred out of the region for treatment, patients who had not received any treatment and cases identified from the death certificate only. Patients who were treated in Wessex but were not residents were excluded from the study. Information including details of presentation, diagnosis, staging and management was recorded and patients were followed-up for 5 years to determine local recurrence and survival.

We analysed 5-year all-cause survival data for all patients staged as Dukes A, B, or C, or ‘D’, but excluded from analysis those where individual data items were not available: principally, these were stage data and data on number of nodes removed. The group was stratified into those with 0–4 nodes identified, those with 5–10 identified, and those with >10 identified. We made univariate survival comparisons using Kaplan–Meier curves and the log rank test for all cases combined and for each stage group.

Cox regression was used to estimate the effect of lymph node count on survival adjusted for possible confounders. These were identified a priori from previous studies and from biological inference (Rothman, 1986). Those considered were age group (<65 years; 65–74; 75–84; 85+), Dukes' stage, site of tumour (right, up to and including splenic flexure, left, and rectal), histological subtype (classified as adenocarcinoma or ‘other’) and receipt of postoperative chemotherapy. In addition, the effect of different surgical expertise was entered into the model by using the proxy measure of average number of nodes removed by surgical firm. The linear effect of continuous variables was tested by including quadratic terms in the regression models. The assumption of proportional hazards was tested using Schoenfeld residuals. Interaction was tested using likelihood ratio tests.

For a sample of our population, we had data on degree of lymphocytic infiltration of the primary tumour as reported by the examining pathologist: infiltration is reported as being either ‘mild’ or ‘prominent’. Further analysis was undertaken on this sample including a comparison of survival by number of nodes identified and a comparison of survival by degree of lymphocytic infiltration. The mean and median numbers of nodes identified in the mild and prominent lymphocytic infiltration groups were compared and the significance of the difference tested using a Mann–Whitney *U* test.

## RESULTS

Of the 5176 cases in the original data set two were found to be anal cancers that had been wrongly included. Of the remaining 5174, 688 had no stage data, and 1398 had no data on number of nodes examined. Together, these cases accounted for 1473 cases excluded from analysis. Further cases were excluded because of missing data on possible confounders, including age, site of tumour, histological type and receipt of chemotherapy, or because they had zero survival or apparent negative survival. Following exclusions, 3592 cases remained in our data set, 403 staged as Dukes' A, 1458 Dukes' B, 953 Dukes' C and 778 Dukes' ‘D’. Table 1 gives the number (%) of study participants according to age group, gender, presentation, tumour site, tumour histology, number of lymph nodes identified and whether or not postoperative chemotherapy was received, by Dukes' stage group. The proportion of tumours identified as adenocarcinomas is lower than expected, implying a small degree of coding error. However, a significant survival advantage is still present for the adenocarcinoma group compared to those described as ‘other’ (*P*=0.007).

**Figure 1:**
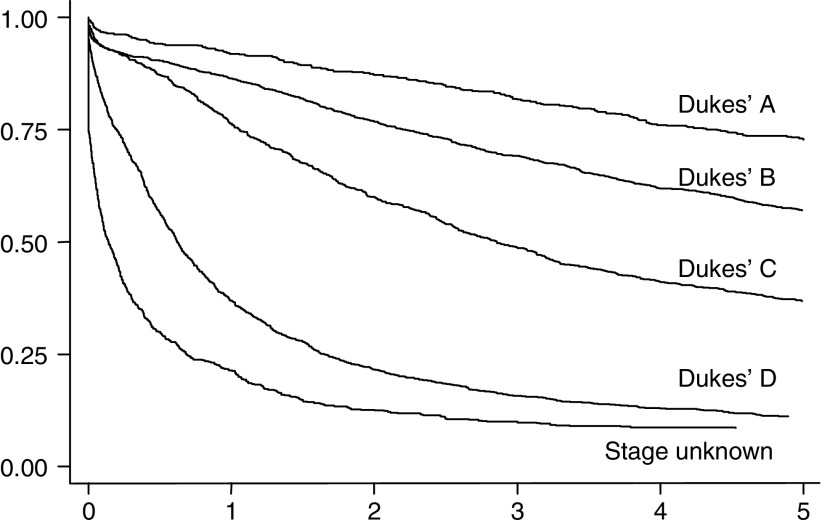
Five-year survival, 5176 cases, by Dukes' stage.

Figure 1 shows Kaplan–Meier curves for all four Dukes' stages and for those with unknown stage. The pattern shows survival decreasing as Dukes' stage moves from A to D, with the worst survival in those with unknown stage. This confirms our anecdotal observation that, in the UK at least, formal staging is often omitted in patients presenting with very advanced disease.

**Figure 2:**
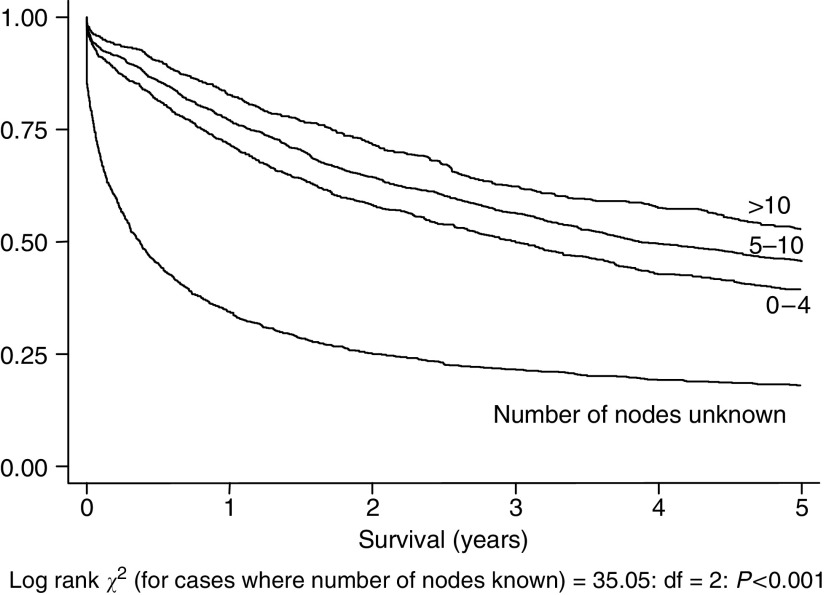
Five-year survival, 5176 cases, by number of nodes examined by pathologist (log rank statistic and statistical significance calculated only for those cases (*n*=3592) where number of nodes examined is known).

Figure 2 shows Kaplan–Meier curves for the three lymph node groups and for those with unknown number of nodes. Again, survival in this last group is associated with very poor survival. The associated log rank statistic is calculated on those groups where data on number of nodes is known. There is a significant survival advantage associated with a larger number of nodes identified across the whole population studied (log rank=35.05, df=2, *P*<0.001).

The assumption of proportional hazards between lymph node count groups was not violated (patients classified as: Dukes' A *P*=0.145; Dukes' B *P*=0.656; Dukes' C *P*=0.309; Dukes' D *P*=0.516). There was no evidence of interaction between lymph node count group and treatment by chemotherapy (patients classified as Dukes' A *P*=0.150; Dukes' B *P*=0.816; Dukes' C *P*=0.746; Dukes' D *P*=0.123). Neither was there evidence of significant interaction between lymph node count group and age group (*P*=0.552), Dukes' stage (*P*=0.905) nor histological tumour type (*P*=0.325).

**Figure 3:**
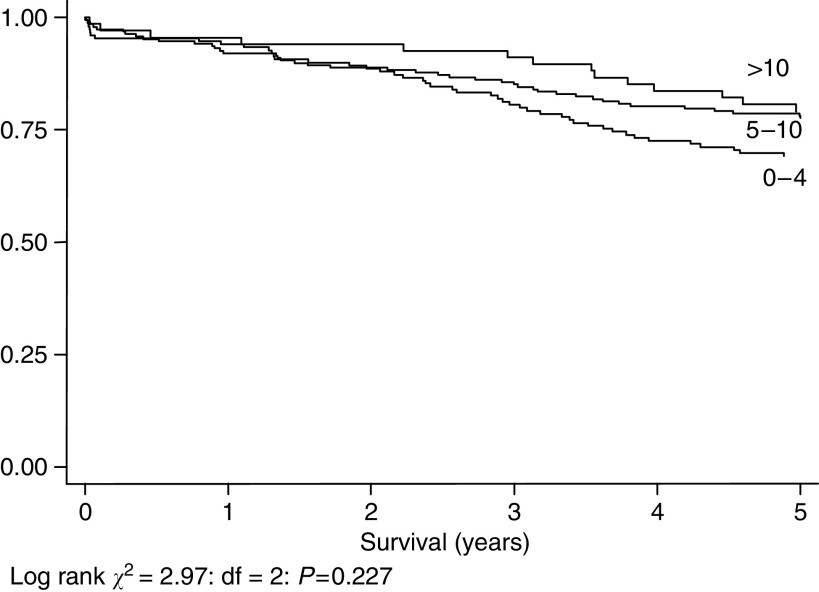
Five-year survival, 403 cases staged as Dukes' A, by number of nodes identified.

**Figure 4:**
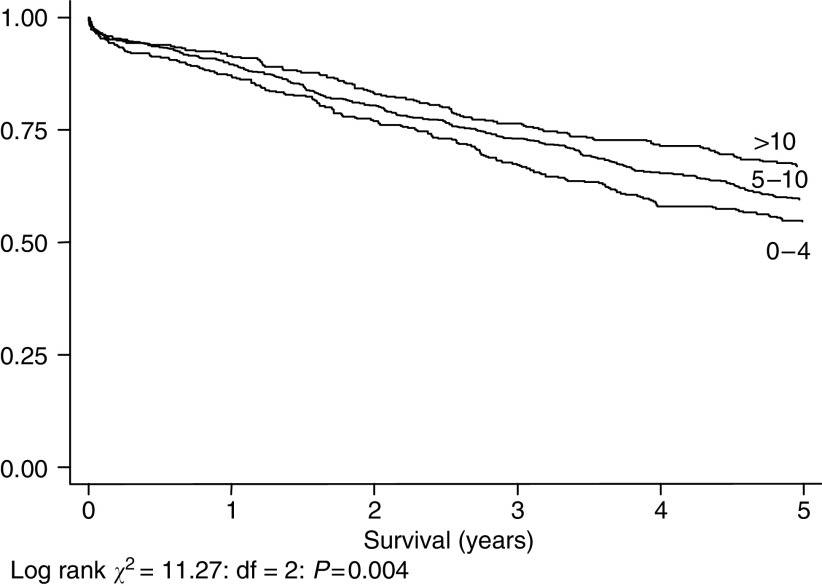
Five-year survival, 1458 cases staged as Dukes' B, by number of nodes identified.

**Figure 5:**
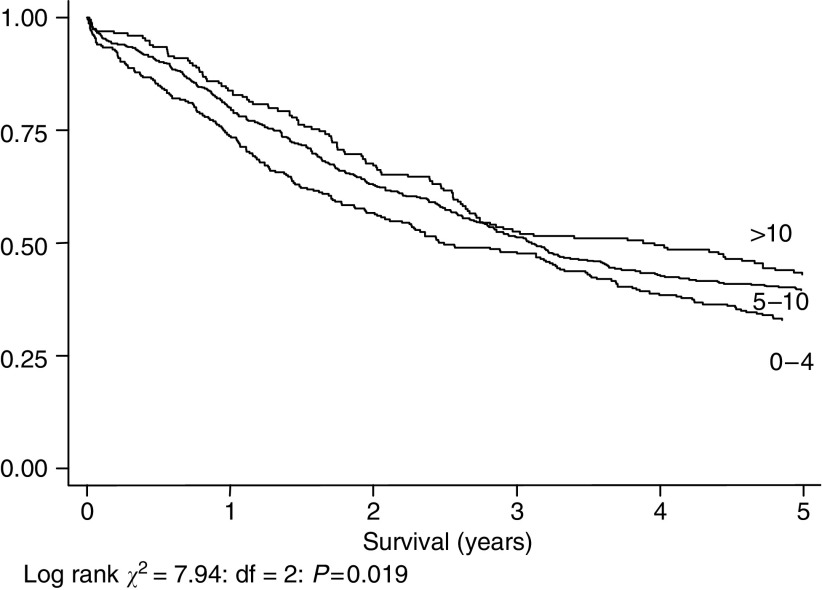
Five-year survival, 953 cases staged as Dukes' C, by number of nodes identified.

**Figure 6:**
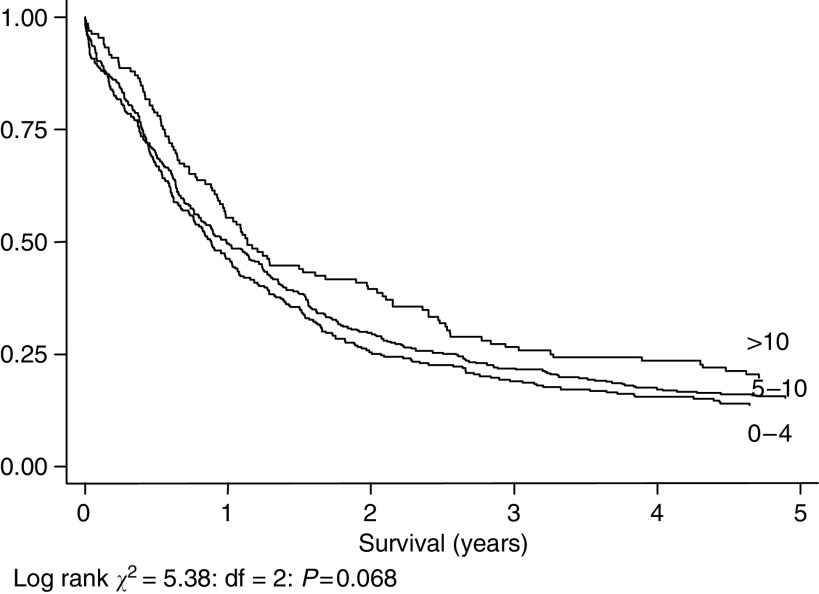
Five-year survival, 778 cases staged as Dukes' D, by number of nodes identified.

Figures 3, 4, 5 and 6 show Kaplan–Meier curves and associated log rank statistics for Dukes A, B, C and D groups, respectively. Within each group a survival advantage is associated with higher numbers of nodes identified, although these differences only achieve statistical significance in the groups containing the largest numbers of cases, Dukes B (*P*=0.004) and Dukes' C (*P*=0.019).

**Figure 7:**
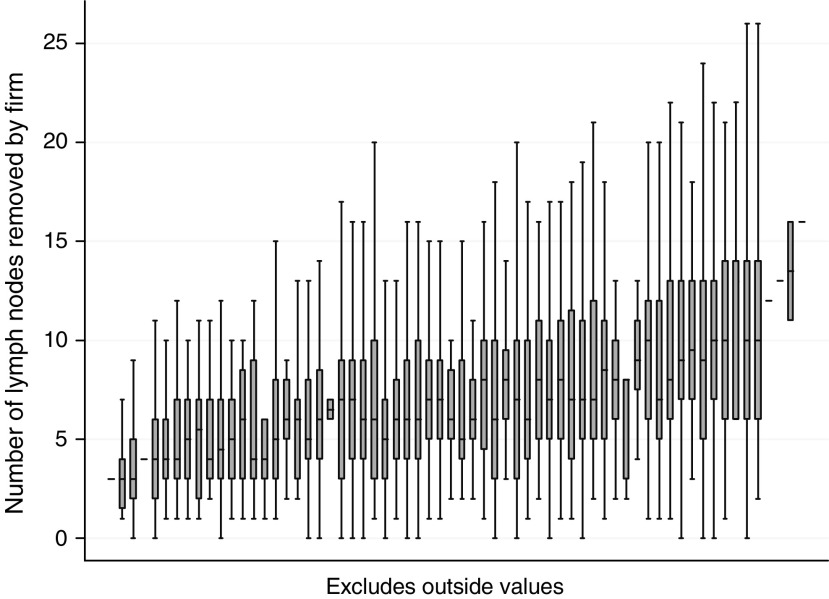
Box and whisker plots, for each surgical firm, showing the distribution of numbers of lymph nodes identified by pathologists in tissue removed during surgery, arranged by the number of procedures undertaken by the surgeon over the period of the study. For the sake of visual clarity outliers and extremes are not shown. Note that the plots are arranged in order of the average (mean) number of nodes removed by firms, but the scale is not linear.

Table 2 shows results of Cox regression analysis for the multi-variable model. Age was categorised since there was evidence that the effect of age was not linear. The survival advantage noted for greater number of nodes noted persists when Cox regression is used to control for confounders. The Hazard Ratio for the 5–10 node group compared to the 0–4 node group is 0.88 (95% CI 0.80–0.98, *P*=0.018) and that for the >10 group compared to the 0–4 group is 0.78 (95% CI 0.68–0.89, *P*<0.001), indicating significantly reduced risk of death in the 5–10 and >10 node groups. The average number of nodes removed by surgical firm was not a significant predictor of survival in this multivariable model. Figure 7 shows box and whisker plots, for each surgical firm, of the distribution of numbers of lymph nodes identified by pathologists in tissue removed during surgery: plots are arranged by the number of procedures undertaken by the surgeon over the period of the study. The variation in numbers of nodes found is greater within firms than the variation between them.

**Figure 8:**
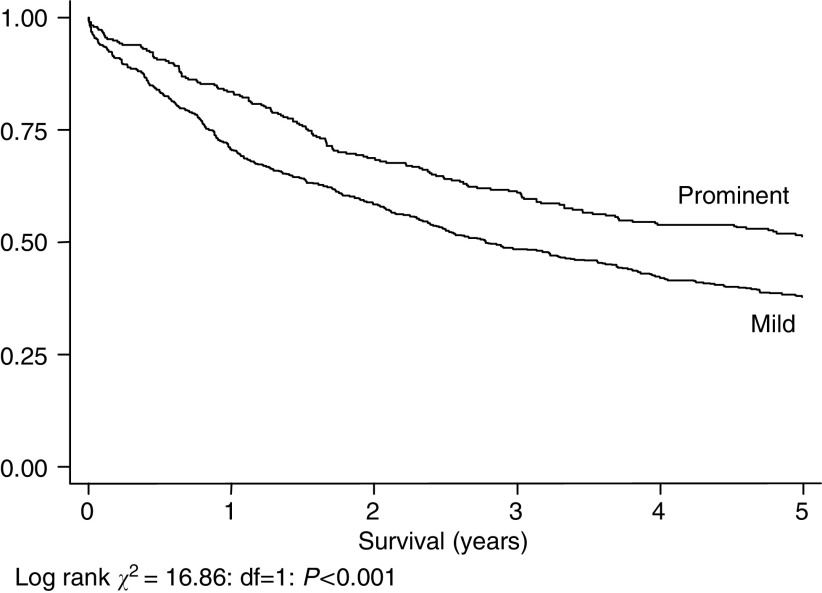
Five-year survival, 890 cases with data on degree of lymphocyte infiltration into primary tumour, by degree of lymphocytic infiltration (prominent, *n*=297: mild, *n*=593).

The overall pattern of greater survival with more nodes identified persists in the sub-group where data on lymphocytic infiltration were available, although it does not reach statistical significance. Figure 8 shows a significant survival advantage within this group in those with prominent rather than moderate lymphocytic infiltration into the primary tumour (Log Rank Statistic=16.86: df=1: *P*<0.0001). The median (interquartile range) number of nodes in the prominent (*n*=297) and mild (*n*=593) lymphocytic infiltration groups is eight (5–11) and seven (4–7) respectively: this difference is significant (Mann–Whitney *U* statistic 79 252.5; *P*=0.015).

Table 3 shows the distribution of numbers of nodes between the two lymphocytic infiltration groups: the ‘prominent infiltration’ group contains no cases where no nodes were found at pathological examination, and in only 23.3% of cases were fewer than five nodes found at examination. By contrast, fewer than five nodes were found in 32.5% of the ‘mild infiltration’ group.

## DISCUSSION

The American humorist Will Rogers once joked that the move of many residents of Oklahoma to California was increasing the average IQ in both places. An analogous explanation for the apparent linkage between extent of pathological examination and cancer survival was first proposed by Feinstein in 1985, and named ‘stage migration’; the argument is that in a centre where pathologists make a greater effort to identify lymph nodes it is more likely that patients with nodal involvement will be staged accurately as being in stage C (Feinstein *et al*, 1985; Poller, 2000). This would have the effect of apparently improving survival in patients in stage B by excluding patients with a worse prognosis. At the same time, these patients might improve survival amongst Stage C patients, as they are likely to be better prognostically than those graded as Dukes' C by pathologists who do not make such an effort. It can be expected, therefore, that the number of lymph nodes identified by a pathologist following surgery for colorectal cancer predicts long-term survival in both stage B and stage C disease. In our study, however, the pattern is seen in the population as a whole, and is similar within each individual stage group, although significance is reached only in Dukes' B and Dukes' C groups. Several alternative explanations for these findings need to be considered.

First, stage migration. This is the conclusion reached by most authors who have published on the subject, and is a perfectly acceptable explanation for the relationship observed between number of nodes removed and prognosis in Dukes' B and C disease seen in this study. However, had IQ been measured in the combined population of Oklahoma and California this phenomenon could not have been observed, nor become the subject of a joke: there would simply have been no change in IQ associated with population movement. Our study shows a significant survival advantage related to number of nodes removed across the whole study population, without division into stages. This cannot be explained by stage migration alone, as any artefact brought about by incorrect staging is eliminated when all stages are considered together. Stage migration may still contribute to the relationship seen within individual stages, however, except for one: it is interesting to note that a relationship between number of nodes removed and survival, similar in both direction and magnitude to that seen in Stage B and C patients, is also seen in Stage D patients, although this does not attain statistical significance (*P*=0.068). Type II statistical error is the most likely explanation for this, based on the smaller group size. However, as node status misclassification cannot influence whether a patient is placed in stage D, patients being classified thus on the basis of presence of distant metastases, this finding supports, albeit softly, our contention that stage migration cannot be the sole explanation for our findings.

If the artefactual explanation offered by stage migration cannot completely explain our results, we must consider other means by which the number of nodes identified by the pathologist could impact upon survival. Several suggest themselves. First, taking out more lymph nodes may reflect superior technical ability on behalf of the surgeon. Second, the identification of lymph nodes containing tumour may change the management of the patient in some way (e.g. by the giving of adjuvant chemotherapy). Third, it may be that the number of lymph nodes present may relate to other factors affecting survival, principally geographical site of the tumour. Last, lymph node enlargement leading to easier harvesting may in itself be a prognostic factor in the patient's outcome.

The first explanation is the most intuitive: surgeons removing a specimen containing more lymph nodes are performing a more radical and technically accomplished operation than those who have a small harvest of lymph nodes, and the impact of the surgeon on outcome in patients having colorectal surgery is well reported (Meagher, 1999). The traditional and radical approach to colon cancer is to remove the tumour with all its associated nodes up to and including the level of the principal arterial supply, and it is self evident that the more of the mesentery that is removed along with the tumour the more nodes there will be that are available for pathological harvest. However, most surgeons will aim to carry out the same operation on each similar patient they treat, whereas it is apparent from our data that the difference in numbers of nodes removed *within* a surgical firm (i.e. between patients) is greater than that *between* surgical firms. Mathematically, the average number of nodes taken out by a surgical firm is not a significant predictor of survival in this multi-variable model, whereas the advantage conferred by the individual number of nodes removed remains.

Another explanation concerns changes in management brought about by the finding of involved lymph nodes. However, participants in this study were recruited between 1991 and 1994. During that period only a minority of patients in the UK, even in Dukes stage C, had postoperative chemotherapy in the hospitals concerned, and receipt of chemotherapy did not contribute significantly to our regression model.

It is possible that number of nodes is associated with site of tumour, itself a prognostic variable in colorectal cancer. Right-sided tumours have worse survival, principally due to late presentation. Also, rectal tumours are thought by many to have a poorer prognosis (stage for stage) than colonic cancers, and some authors have reported that there are fewer nodes found around the rectum than around other segments of the colon (Maurel *et al*, 1998). Again, however, site of tumour did not contribute significantly to our regression model.

Another possible explanation is based upon the observation that reactive lymph node enlargement is itself a prognostic factor in colorectal carcinoma (Pihl *et al*, 1980). The number of nodes found within a specimen relies on two principal factors: first, the amount of tissue, particularly mesenteric, taken out by the surgeon and, second, the number of nodes that the pathologist is able to find in the specimen. The first of these we have already found not to be a significant predictor of survival in this model. The real explanation for our findings may relate to how easy it is for the pathologist to locate lymph nodes. Tiny lymph nodes in a fatty mesentery can be extraordinarily difficult to locate, whereas large nodes in a thin mesentery are comparatively easy to find. Lymph nodes enlarged because of malignancy are more easily found but, in addition, it is probable that uninvolved nodes enlarged because of reactive change are also more easily found. It is known that immune response to colorectal cancer affects survival: patients who have tumours with a pronounced lymphocytic infiltrate have a good prognosis compared with those who have no such infiltration (Jass, 1986; Fernandez-Acenero *et al*, 2000; Murphy *et al*, 2000; Canna *et al*, 2005). We have shown here that the number of lymph nodes found by the pathologist is increased in those with prominent lymphocytic infiltration of the primary tumour. It may be, therefore, that the number of lymph nodes found is a function of their reactive enlargement. Recent UK guidelines suggesting that a minimum of 12 nodes be examined may be easier to implement in some patients than in others, therefore (National Institute for Clinical Excellence, 2004). It may also be time to consider whether to include an assessment of the degree of lymphocytic infiltration into the primary tumour as part of the minimum data set.

In conclusion, we have demonstrated that the number of lymph nodes harvested in patients with curable colorectal cancer correlates with a better survival, and also with immune reaction to tumour. It is likely that some of the survival advantage noted in this study is due to stage migration, and we do not doubt the value of impeccable surgical technique and pathological examination. However, stage migration alone cannot explain our results, and it may be that biological predetermination is a key factor in differences in patient survival from colorectal cancer (Macdonald, 1951; Jass, 1999).

## Figures and Tables

**Table 1:** Number (%) of study participants according to age group, gender, presentation, tumour site, tumour histology, number of lymph nodes identified and whether or not postoperative chemotherapy was received, by Dukes' stage group

	**Dukes' stage**				
	**A**	**B**	**C**	**D**	**All**
*Age group*					
<65	118 (29)	344 (24)	269 (28)	231 (30)	962 (27)
65–74	158 (39)	483 (33)	311 (33)	270 (35)	1222 (34)
75–84	97 (24)	489 (34)	293 (31)	214 (28)	1093 (30)
85+	30 (7)	142 (10)	80 (8)	63 (8)	315 (9)
					
*Gender*					
Male (%)	216/403 (54)	736/1458 (51)	494/953 (52)	408/778 (52)	1854/3592 (52)
					
*Presentation*					
Elective	390 (97)	1255 (86)	830 (87)	649 (83)	3124 (87)
Emergency	10 (2)	193 (13)	116 (12))	122 (16)	441 (12)
Incidental	1 (0.2)	5 (0.3)	4 (0.4)	4 (0.5)	14 (0.4)
Not known	2 (0.5)	4 (0.3)	3 (0.3)	3 (0.4)	12 (0.3)
					
*Tumour site*					
Right colon	55 (14)	529 (36)	290 (30)	305 (39)	1179 (33)
Left colon	162 (40)	558 (38)	344 (36)	289 (37)	1353 (38)
Rectum	186 (46)	371 (25)	319 (34)	184 (24)	1060 (30)
					
*Histology*					
AdenoCa	374 (93)	1314 (90)	856 (90)	681 (86)	3225 (90)
Other	29 (7)	144 (10)	97 (10)	87 (13)	367 (190)
					
*Number of lymph nodes identified*					
0–4	149 (37)	390 (27)	286 (30)	279 (36)	1104 (31)
5–10	187 (46)	694 (48)	469 (49)	367 (47)	1717 (48)
>10	67 (17)	374 (26)	198 (21)	132 (17)	771 (21)
					
*Postoperative chemotherapy*					
Received	6 (2)	53 (4)	119 (12)	133 (17)	311 (9)
Not received	397 (99)	1405 (96)	834 (88)	645 (83)	3281 (91)
					
*Total*	403 (11)	1458 (41)	953 (27)	778 (22)	3592 (100)

Percentages may not add up to 100% because of rounding.

**Table 2:** Results of Cox regression analysis of survival for the adjusted (multivariable) model

		**Adjusted effect**	
	**Number of deaths in group (% of cases in group)**	**Hazard ratio**	**95% CI**
*Age*			
<65	418 (43)	1	
65–74	643 (53)	1.37	1.21–1.55
75–85	663 (61)	1.80	1.58–2.05
85+	220 (70)	2.27	1.91–2.69
			
*Number of lymph nodes*			
0–4	659 (60)	1	
5–10	921 (54)	0.88	0.80–0.98
>10	364 (47)	0.78	0.68–0.89
			
*Dukes' stage*			
A	105 (26)	1	
B	588 (40)	1.65	1.34–2.03
C	593 (62)	3.25	2.64–4.01
D	658 (85)	7.31	5.92–9.02
			
*Site*			
Left colon	709 (52)	1	
Right colon	674 (57)	1.06	0.95–1.18
Rectum	561 (53)	1.09	0.98–1.22
			
*Post-operative chemotherapy*			
No	1757 (54)	1	
Yes	187 (60)	0.97	0.83–1.14
			
*Histological subtype*			
Other	217 (59)	1	
AdenoCa	1727 (54)	0.84	0.72–0.97
			
*Average number of nodes removed by surgical firm*			
0–7.15	979 (52)	1	
7.23–16	965 (56)	0.97	0.89–1.07

**Table 3:** Distribution of numbers (percentages) of cases by number of lymph nodes identified in 890 cases with data on degree of lymphocyte infiltration into primary tumour, and by degree of lymphocytic infiltration

	**Number of nodes**							
	**0**	**1**	**2**	**3**	**4**	**5–10**	**11+**	**Total**
Prominent infiltration	0 (0)	10 (3.3)	13 (4.3)	17 (5.7)	30 (10)	151 (51)	76 (25)	297
Mild infiltration	6 (1)	30 (5)	34 (5.7)	68 (11.4)	56 (9.4)	250 (42)	149 (25)	593
